# Effects of Light Wavelength on Broiler Performance, Blood Cell Profiles, Stress Levels, and Tibiotarsi Morphology

**DOI:** 10.3390/ani15162372

**Published:** 2025-08-13

**Authors:** Angela Perretti, Victor J. Oyeniran, Jaelen M. Cherry, Rosemary H. Whittle, Zachary Grider, Alexander H. Nelson, Seong W. Kang, Gisela F. Erf, Shawna L. Weimer

**Affiliations:** 1 Department of Poultry Science, University of Arkansas, Fayetteville, AR 72701, USA; 2 Department of Electrical Engineering and Computer Science, University of Arkansas, Fayetteville, AR 72701, USA

**Keywords:** broiler, fear, activity, wavelength, performance, immune function

## Abstract

Lighting influences broiler production, health, and behavior. The objective of this study was to examine the effects of three light wavelengths (White [350–780 nm], Blue [450 nm], and Green [560 nm]) on broiler production, activity, fear, stress, blood cell profiles, and tibiotarsi (tibia) morphology. Day-of-hatch male broiler chicks (N = 600) were housed in pens (N = 12) with one lighting treatment for 42 days. Body weight and feed consumption were recorded on day (D) 0, 14, 28, and 42, and the feed conversion ratio was calculated. The Tonic Immobility test was used to assess the latency (seconds) to right from the testing cradle (D12 and D33). Blood was drawn for leukocyte and plasma corticosterone concentrations (D21 and D41). Accelerometers were attached at 2 and 5 weeks of age to measure activity. On D41, thermal images of the head were taken to measure surface temperatures (eye and beak), the bursa of Fabricius (bursa) was extracted for relative bursa weight, and the right and left tibias were extracted for tibia morphology. After sampling, the remaining broilers were processed. Activity was greatest in Green light at Week 2 (261.17 ± 8.52 m/s^2^, *p* < 0.0001), and activity was lowest in White light at Week 5 (98.99 ± 8.52 m/s^2^, *p* < 0.0001). Broilers under Blue light had lower concentrations of lymphocytes (8.62 ± 0.40 × 10^3^ cells/µL, *p* = 0.01) and T cells (7.16 ± 0.33 × 10^3^ cells/µL, *p* = 0.008) compared to White light. Breast yields were greatest (26.89 ± 0.13%, *p* = 0.01) in the White treatments compared to Green and Blue treatments. These results suggest that blue light may negatively impact immune health, while green light increases activity, without decreasing production performance, and white light can improve carcass traits. Therefore, lighting color can be strategically used to target welfare or production goals.

## Simple Summary

Colored lighting can be used as a non-invasive tool to improve broiler health and welfare by influencing physiology, behavior, and performance. This study examined the effects of white, green, and blue light on broiler production performance, tibiotarsi morphology, behavior, leukocyte profiles, and stress indicators. Overall, light color did not significantly affect performance, fear and stress levels, and tibiotarsi morphology. Broilers under Blue light had lower blood leukocyte profiles, Green light increased activity, and broilers under White light had the highest breast meat yields. This study suggests that lighting wavelengths affect activity, immune health, and carcass traits of broilers.

## 1. Introduction

Lighting plays a critical role in broiler production, health, and behavior. Recent trends in broiler management have increasingly focused on lighting solutions for improving bird welfare [1,2]. Although light color has been shown to have the potential to impact broiler welfare and performance [3,4,5], the underlying mechanisms are not well understood. Conflicting results from lighting research may be due to the sensitivity of poultry vision and/or variations in lighting characteristics (e.g., intensity, wavelength, photoperiod) [6]. Gradient lighting schedules are being adopted in commercial broiler houses after showing positive effects on poultry production [7], but the use of different light colors (wavelengths) has not produced consistent results. Therefore, further research is needed to elucidate the effect of light wavelength on broiler performance and welfare.

Broilers are inherently affected by light due to their photosensitive organs and glands (e.g., retina, hypothalamus, pineal gland) [8,9], and their unique perception of light wavelengths [5]. Light must travel through layers of the body (e.g., feather, skin, bone, brain) to reach the hypothalamus and pineal gland [8,9]. The depth of light penetration into the brain and effect on the endocrine system is determined by its wavelength [9], which is directly related to its energy capacity [9]. Longer wavelengths (e.g., red, orange, yellow) possess greater energy and penetrate more deeply into brain tissues, increasing the likelihood of it reaching the hypothalamus [8,9,10]. Shorter wavelengths (e.g., blue, green, violet) are more readily absorbed by superficial tissues, which may not reach deeper brain structures as effectively [8]. Once light reaches the hypothalamus, it stimulates the release of hormones (e.g., corticosterone and serotonin) that regulate circadian rhythm [5]. As a result, light plays a critical role in modulating reproduction, behavior, and homeostasis.

Previous research has shown that light wavelengths, presented singularly or in repeated patterns, affect broiler performance [5,11,12,13]. Green light has been shown to improve body weight gain as early as day D3 [12], while blue light has been shown to increase the eating behavior of broilers [14]. Studies have evaluated the effects of switching light colors for broilers at different ages. One study found that transitioning from blue to green light increased eating and has been shown to improve body weight gain as early as D3 [12]. Another study found blue light increased eating behavior, yet this did not translate to greater body weight [13]. Some studies have found no effect of light color on growth performance [5,11,12,13], yet broilers were found to drink the most in white light in one study [11] and eat the most in green light in another study [5]. In summary, consummatory behaviors do not always translate to production and performance differences.

Light wavelength has also been shown to impact broiler carcass traits [4,13,14,15]. White, blue, green, and sequentially colored lighting patterns have been examined for carcass, breast, thigh, and drumstick weights in various studies [4,15,16,17]. Broilers raised in white, blue, green, and red light were observed to have greater breast, thigh, and carcass weights under blue light [15], heavier abdominal fat in blue-green light [17], heavier breast muscle in blue, green, and sequential blue then green light, and heavier thigh and leg muscles in sequential green then blue light [4]. Colored light treatments of white, blue, red, and yellow did not have an impact on carcass, breast, thigh, or drumstick yields in one study [18], while broilers raised in white, blue, green, red, or blue-green light had lower breast yields in white light compared to blue light in another study [19]. Blue light has been found to increase carcass, breast, and thigh weights in some studies [5,15,16,17], while others did not find differences [18]. In some studies, an increase in body weight translated to greater parts weights [15,16]. Taken together, blue light has been shown to have a greater effect on carcass traits than other wavelengths, though the effects of different light wavelengths can vary depending on the lighting regimen and experimental conditions.

Broiler behavioral repertoires are affected by light color, which has been demonstrated across several studies [5,11,13,14,20,21]. Exposure to blue light has been associated with increased resting behaviors, green light promoted comfort behaviors, and white light increased walking activity [11,13]. Prayitno et al. [11] found that broilers raised in green light spent more time sitting compared to broilers reared in white, blue, and red light. In contrast, Sultana et al. [20] found that broilers raised in blue light sat more compared to white, green, yellow, and combinations of yellow-green and green-blue light. In one study, broilers raised under white, blue, or green light did not have behavioral differences, but the interactions of light color, genotype, and sex revealed that EPMx708 females raised in green light ran more compared to all interactions [5]. Some responses to light wavelengths have been associated with negative welfare indicators; for example, green light increased activity and was also linked to increased fearfulness [5]. In another study, blue light was shown to reduce activity and increased leg health issues [22]. Although results vary, it has been shown that green light promoted activity and may serve as a tool to improve broiler welfare.

Light not only affects the behavior and performance of broilers, but also immune responses [23,24]. For example, sequential presentation of green to blue light has increased antibody responses to bovine serum albumin and Newcastle disease virus, the mitogen-stimulated proliferation of peripheral blood T lymphocytes, the lipopolysaccharide (LPS) stimulated proliferation of peripheral blood B lymphocytes, and serum IL-2 concentrations compared to single-color exposure to white, blue, green, or red light [23]. Xie et al. [24] examined mitogen-stimulated proliferation of peripheral blood T lymphocytes in response to white, blue, green, and red light and found that the proliferation of peripheral blood T lymphocytes was greatest at D21 and D49 in the white light group [24]. Increased antibodies and lymphocytes are important to disease and infection resistance and overall health [25], which can be modulated by light wavelength.

The aim of this study was to examine the use of three light wavelength treatments (White [350–780 nm], Blue [450 nm], and Green [560 nm]) on broiler production and processing performance, tibiotarsi (tibia) morphology, behavior (activity and fear), immune blood cell profiles, and stress (corticosterone, relative bursa weights, facial surface temperatures). It was hypothesized that broilers in the White treatment would have greater activity, lower performance, and greater fear and stress responses than broilers under Blue and Green light.

## 2. Materials and Methods

This experiment was completed from January to February 2024, and the University of Arkansas Division of Agriculture Institutional Animal Care and Use Committee (IACUC) approved all procedures. The study took place at the Arkansas Agricultural Experiment Station (AAES) Poultry Research Farm in Fayetteville, AR.

### 2.1. Housing and Facilities

Day-of-hatch male Cobb 500 by-product chicks (N = 600) were transported from a commercial hatchery (Cobb-Vantress, Siloam Springs, AR, USA) to the AAES Poultry Research Farm, and all chicks were randomly distributed across 12 environmental chambers (pens) (N = 50 chicks/pen). The pen dimensions were 2.4 m × 3.6 m and contained clean wood shavings at a depth of 7 cm, two gravity-fed hanging feeders, and two lines of nipple drinkers. Feed and water were provided ad libitum. Diets were formulated to meet Cobb recommendations [26] and were fed as starter crumble from D0–14, pelleted grower from D14–28, and pelleted finisher from D28–41. The trial lasted 42 days. Pen temperatures were set to 32.2 °C on D0 and decreased incrementally to 22.2 °C by D20 until the end of the study. Daily ambient temperatures within each pen were measured with an infrared thermometer (IRT657, General Tools & Instruments LLC, Secaucus, NY, USA). The photoperiod was 23L:1D on D0, and decreased incrementally to 18L:6D on D17, then 16L:8D from D31 until the end of the study.

### 2.2. Experimental Design

**Figure 1:**
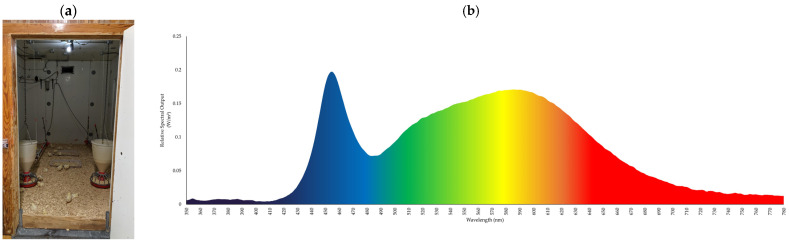

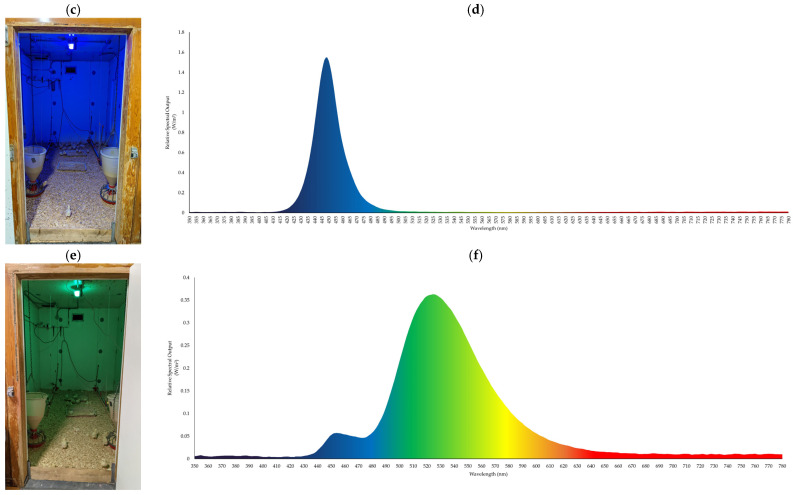
Spectra graphs displaying the relative spectral output (W/m^2^) for each individual light color treatment wavelength and pens: (**a**,**b**) White light at 350–780 nm, (**c**,**d**) Blue light at 450 nm, and (**e**,**f**) Green light at 560 nm. Note *y*-axis scales differ.

The study followed a complete block design with light treatments applied to a pen, for a total of four blocks. Broilers were assigned to three lighting wavelength treatments: White (350–780 nm), Blue (450 nm), and Green (560 nm) (Figure 1) across the 12 pens (N = 4 pens/treatment). The White light treatment served as a control representing lighting conditions in commercial broiler houses. Lights were mounted in the center of the ceiling in each pen. The Blue and Green lights were LED dome lights (11 W) (NatureDynamics DOME lighting, ONCE by Signify, Eindhoven, The Netherlands), and the White lights used a dimmable LED light bulb (9 W) (Greenlite 60 W Equivalent LED, Anaheim, CA, USA). Prior to the placement of chicks, the Blue and Green lighting photoperiods and intensities were programmed with a mobile device application (Interact Agriculture, Signify, Eindhoven, The Netherlands), which automatically changed the Gallilux and photoperiod throughout the study. The Gallilux for all treatments incrementally changed from 20 Gallilux (D0 to D6), to 15 Gallilux (D7), to 10 Gallilux (D13), to 5 Gallilux (D19 to D42). A spectrometer (Hato One Light Spectrum Meter, Hato Agricultural Lighting, Sittard, The Netherlands) was used to record light measures (wavelength, lux, and Gallilux) in each pen prior to chick placement. Additional measurements were taken during manual adjustments to the White light treatment to ensure that Gallilux matched both those of the Blue and Green light treatments. Gallilux and photoperiod in the White light were controlled by a manual wall dimmer and a 24 h dial timer (24-Hour Mechanical Time Switch Model: T101, Intermatic, Libertyville, IL, USA) and were adjusted according to the light schedule and photoperiod changes in the treatment groups. Gallilux measurements for the White lights were taken from the height of a broiler (5 cm to 15 cm), depending on the age of the broiler, and at the center of the pen for each manual White light change. Blue and Green lights were also measured at each Gallilux change to verify consistency across all treatments. The irradiance (W/m^2^) was calculated using the spectral power at each individual wavelength (350 to 780nm) for each treatment when taking the light measures (White = 0.033 W/m^2^, Blue = 0.040 W/m^2^, and Green = 0.030 W/m^2^).

### 2.3. Data Collection and Sampling

#### 2.3.1. Production

All birds were weighed by pen at each diet phase change (Starter [D14], Grower [D28], Finisher [D41]), and individual body weight (BW, kg) was calculated from the total pen weight divided by the total number of birds in each pen. Feed consumption was recorded at each diet phase change, and the feed conversion ratio (FCR) was calculated by dividing the cumulative feed consumption by the total pen weight at each dietary phase change. Throughout the study, mortalities were recorded and used in the calculation for adjusted FCR.

#### 2.3.2. Tonic Immobility

Birds were selected randomly from each pen on D12 (3 birds/pen, N = 36 birds) for the Tonic Immobility (TI) test, and the same birds were tested on both ages (D12 and D33). After testing on D12, each broiler was marked with blue livestock paint (Quik Shot Spray Paint for Livestock Marking, LA-CO Industries, Elk Grove Village, IL, USA). During the test, each bird was removed from their home pen and carried to the TI testing area at the far end of the facility, about 6 m away. The bird was placed dorsally into a wooden V-shaped cradle, in which the induction period began. The test cradle was widened at D33 to account for the larger size of the test birds. The experimenter placed light pressure on the chest of the bird with one hand and covered the face of the bird with the other hand for 15 s. Two different experimenters handled the test birds during the TI test at both ages. If the test broiler did not right itself (i.e., by lifting itself up completely out of the cradle or standing up in the cradle) within 10 s of induction, the test continued for a maximum of 300 s. If the broiler righted itself within 10 s, the experimenter attempted to induce TI again for a maximum of three attempts. The latency (s) to right from the testing cradle and the number of attempts to induce immobility for each broiler were recorded.

#### 2.3.3. Stress

On D21 and D41, blood was collected from birds that did not undergo TI testing (N = 4 birds/pen, 48 birds). Blood (at least 1.5 mL) was drawn from the brachial vein using heparinized 3 mL syringes with 2.54 cm × 21-gauge needles. Time of day that each blood sample was drawn was recorded. From each sample, whole blood (20 µL) was used for direct immunofluorescent staining and fluorescence activated flow cytometry (BD Accuri™ C6 Plus Personal Flow Cytometer, San Jose, CA, USA) to determine blood cell profiles (T cells, B cells, lymphocytes, heterophils), total white blood cells (WBC), red blood cells (RBC), and thrombocytes following procedures described by Santamaria et al. [27]. Two stains were used that consisted of CD41/61-FITC and CD45-SPRD (Stain A) and Bu-1-FITC and CD3-SPRD (Stain B). For each sample, a total of 300,000 cells/sample were acquired and analyzed using software (FlowJo v.7, BD Biosciences, Ashland, OR, USA). Heterophil–lymphocytes ratios (HLR) and T:B cell ratios (TBR) were calculated by dividing the concentration of heterophils by the concentration of lymphocytes and dividing the T cell concentrations by the B cell concentrations, respectively.

The remaining whole blood was centrifuged at 3500× *g* RPM for 3 min to separate plasma, and the plasma was stored at −80 °C. Plasma corticosterone (CORT) levels were measured using an enzyme-linked immunosorbent assay (ELISA) (DetectX^®^ Corticosterone ELISA, Arbor Assay, Ann Arbor, MI, USA) at a dilution factor of 1:20. The ELISA plates were read using the BioTek Gen5 software (BioTek Gen5, Agilent, Santa Clara, CA, USA) at 450 nm.

Immediately prior to blood collection on D41, thermal images of the head of each bird were taken within 15 to 20 s of removal from the home pen using an infrared thermal camera (Ti480 Pro Thermal Camera, Fluke^®^, Everett, WA, USA) with a background temperature of 22 °C, emissivity of 0.95, and transmission of 100%. Thermal images were analyzed using Fluke software (SmartView Classic v4.4, Fluke^®^, Everett, WA, USA). An ellipse tool was used to measure the pixels representing the minimum, maximum, and average surface temperature of the eye and beak regions of the birds.

Following blood collection on D41, birds were euthanized by carbon dioxide (CO_2_) inhalation. The bursa of Fabricius (bursa) was extracted from each bird and stored in 50 mL conical tubes that contained 10% buffered formalin. The bursae were weighed, and the relative weight to body weight was calculated (% BW).

#### 2.3.4. Activity

Activity was measured using tri-axial accelerometers (X3 Accelerometers, Axivity, York, UK). Accelerometers recorded acceleration in x, y, and z axes at a frequency of 100 Hz with a ±8 g range and 13-bit resolution (i.e., approximately 0.002 g). Each logger weighed 16 g and was attached to randomly selected birds that did not undergo TI testing or blood sampling (N = 1 bird/pen, 12 birds) on D11 to D14 and D38 to D41. Accelerometers were secured using two elastic bands (12 mm × 20.3 cm) that were taped to each side of the accelerometer and tied snugly around each wing, allowing for one finger to fit between the elastic band and the bird. Birds were marked with pink livestock paint (Quik Shot Spray Paint for Livestock Marking, LA-CO Industries, Elk Grove Village, IL, USA) after accelerometer attachment. Data from D12 to D13 (Week 2) and D39 to D40 (Week 5) were analyzed to allow for a 24 h acclimation period and ensure 48 h of usable data. Data were downloaded from the accelerometers using Open Movement v.1.0.0.45 (Open Movement, Axivity, York, UK), and activity metrics from accelerometers were determined using the magnitude of acceleration at each sample. Units for acceleration are reported as m/s^2^.

(1) $$\sqrt{{x_{t}}^{2}+{y_{t}}^{2}+{z_{t}}^{2}}$$

All measurements were taken in approximately the same geological location, and therefore, the gravitational component of acceleration is nearly consistent (approximately 9.8 m/s^2^). To remove this constant, we modified the activity metric by subtracting a constant 1g from each sample [28].

(2) $$A_{t}=\sqrt{{x_{t}}^{2}+{y_{t}}^{2}+{z_{t}}^{2}}-9.8$$

Acceleration is a force and is related to the caloric expenditure through the work-energy theorem. The forces measured by the accelerometers are proportional to the displacement over a unit time. Therefore, the daily energy expenditure of the broiler is proportional to the sum of the magnitude of acceleration over time.

(3) $$TDEE\;\propto \sum A_{t}$$

Based on this rationale, we report the acceleration data as the sum of the magnitude of acceleration on a minute-by-minute basis, trimming the first and final minute of each session so that each datapoint contains approximately 6000 measurements (i.e., 100 Hz times 60 s). The summation of the magnitude over time results in a unit of measurement of meters per second squared times the number of samples. This is a non-physical unit of measure that can be used as a comparative metric as a proxy for comparing total daily energy expenditure. After the analysis was complete, data were condensed for readings every hour.

#### 2.3.5. Tibiotarsus Morphology

On D41, the accelerometer birds (N = 1 bird/pen, 12 birds) were euthanized, and left and right tibiotarsi (tibias) were removed, cleaned, and stored at −20 °C. For each bird, measurements from the left and right tibias were averaged. Using a digital caliper and methods adapted from Magnaterra et al. [29], the following measures were recorded: tibia length (mm), angle of proximal head (°), distal and proximal head widths (mm), widths at 90%, 75%, 50%, 25%, and 10% of total lengths (mm), and condyle depths for proximal and distal head (mm). The angle of the proximal head was measured in images using ImageJ software (ImageJ v. 1.54p, NIH, Bethesda, MD, USA). Tibias were also processed for bone ash content (%) over four days. On the first day, tibias were thawed and air dried in crucibles under a fume hood for 24 h. On the second day, the dried tibias were weighed and then placed inside a drying oven set at 43.3 °C for 24 to 48 h. On the third day, the tibias were taken out of the drying oven, reweighed, broken into two pieces per crucible, and placed inside a furnace set to 600 °C for 24 h. On the fourth day, the tibia ash was removed from the furnace and weighed. Tibia ash percentage was calculated as follows: (ash weight/dry bone weight) × 100.

#### 2.3.6. Processing

On D42, all remaining birds (N = 482) were processed at the University of Arkansas Pilot Processing Plant following a 10 h overnight feed withdrawal. Individual body weights (g) were recorded prior to hanging. Broilers were stunned with an electric stunning bath (11 V, 11 mA for 11 s) and exsanguinated before scalding (55 °C for 120 s). Carcasses were de-feathered, neck and feet were removed, and carcasses were eviscerated (Baader Linco 1396, BAADER LINCO, Inc., Kansas City, KS, USA). Fat pad and carcass weights (g) were collected, and carcasses were weighed (g) again after chilling. Carcasses were manually deboned into breast filets (Pectoralis major), tenders (Pectoralis minor), wings, leg quarters, and rack with skin. Part yields were calculated as a percentage (%) of the chilled carcass weights. Breast filets were scored for woody breast, a myofiber degeneration of the breast tissue that causes rigid breast muscle [30], on a 3-point scale (0 = normal, 1 = moderate, 2 = severe) adapted from a 4-point scale described by Tijare et al. [31], in which a score of 2 was a combination of the criteria for scores 2 and 3.

### 2.4. Statistical Analysis

Statistical analyses were conducted using JMP Pro 17 (SAS Institute Inc., Cary, NC, USA). Data were tested for normality using the Distribution platform. A one-way ANOVA was used to determine the effect of lighting treatment on production (BW, FCR, and mortalities), relative bursa weights, thermal images, processing performance, and tibia morphology. Two-way repeated measures ANOVAs were conducted to determine the effects of age, treatment, and age × treatment interactions on TI, CORT, blood cell populations, and activity. Time of day was included in the model as a covariate for CORT to account for circadian rhythm. Data were tested for normality using the Distribution platform. Significant LS means were separated post hoc with Tukey’s HSD. Significance was determined at *p* ≤ 0.05, and a tendency (trend) at *p* < 0.10. Data are reported with main effect *p*-values (denoted as *p*) and, if significance was found, pairwise comparison *p*-values (denoted as *p*) are reported for each significant difference between the two treatment means.

## 3. Results

### 3.1. Performance

Light did not affect BW or FCR at any phase (Table 1). Mortalities (%) during starter (White, 2.52%; Blue, 6.00%; Green, 2.50%), grower (White, 2.53%; Blue, 2.15%, Green, 1.01%), and finisher (White, 3.18%; Blue, 3.82%; Green, 3.07%) were not different across treatments. Light had a tendency to affect BW in the grower phase (*p* = 0.06), with broilers in the Blue treatment (1.86 ± 0.01 kg) weighing less than broilers in the White treatment (1.90 ± 0.01 kg).

### 3.2. Tonic Immobility

**Figure 2:**
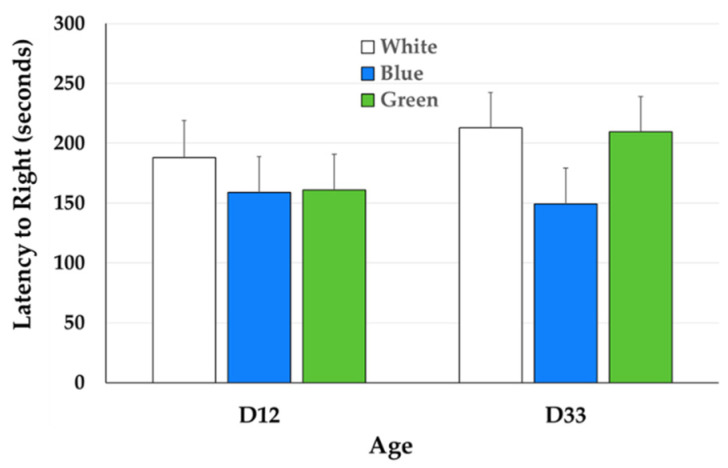
Latency to right (seconds) during the Tonic Immobility test for 12- and 33-day old broilers raised under white, blue, and green light.

Most birds required only one induction attempt for Tonic Immobility (TI): 73.24% (52 birds) were induced on the first attempt, 22.54% (16 birds) on the second, and 4.22% (3 birds) on the third (data not shown). One broiler could not be induced within three induction attempts and was excluded from analysis. The main effects of treatment, age, and interaction were not significant for the latency to right during the TI test (Figure 2).

### 3.3. Blood Cell Profiles

Differences in main effects of treatment were found for concentrations of lymphocytes (*p* = 0.01), with broilers in the Blue treatment having lower (8.62 ± 0.40 × 10^3^ cells/µL) concentrations than birds in the White treatment (10.28 ± 0.40 × 10^3^ cells/µL) (Table 2). Similar main effects of treatment were also found for concentrations of T cells (*p* = 0.009), with the Blue treatment resulting in lower (7.16 ± 0.33 × 10^3^ cells/µL) concentrations than the White treatment (8.64 ± 0.33 × 10^3^ cells/µL) (*p* = 0.008), and the T cell concentrations in the Green treatment (8.16 ± 0.33 × 10^3^ cells/µL) were not different from those of the Blue or White treatment (Table 2). Main effects of age were found for the concentrations of heterophils, lymphocytes, T cells, B cells, TBR, WBC, and RBC. Concentrations of heterophils, lymphocytes, T cells, B cells, WBC, and RBC were higher at D41 (*p* < 0.0001), while the TBR was lower at D41 (*p* = 0.04).

### 3.4. Stress Physiology

There were no significant effects of treatment, age, and their interaction on plasma CORT (ng/mL) on D21 or D41. Across all treatments, the mean CORT levels were 2.31 ng/mL at D21 and 2.29 ng/mL at D41. Relative bursa weights (%BW) did not differ for broilers across treatments, with birds averaging 0.10%. There was no effect of treatment on eye and beak surface temperatures, but there was a tendency for birds in the White treatment to have lower average (*p* = 0.09) and maximum (*p* = 0.07) eye temperatures than birds in the Green treatment (Table 3).

### 3.5. Activity

**Figure 3:**
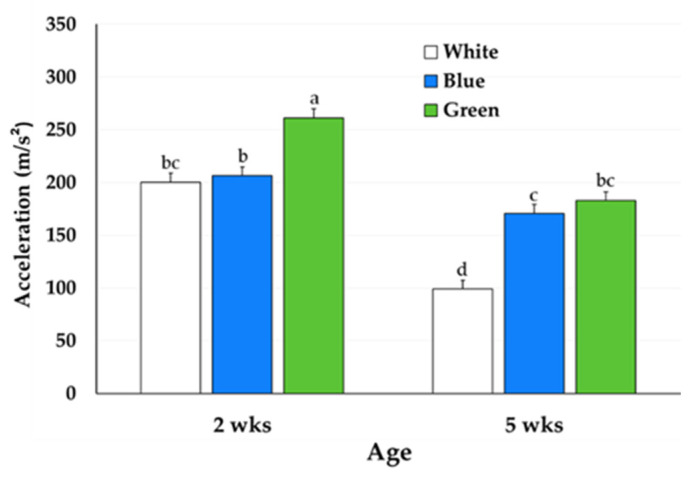
Activity (m/s^2^) of broilers raised under White, Blue, and Green light during week 2 (D12–D13) and week 5 (D39–D40) of age. ^a–d^ Bars not sharing the same letter indicate a significant difference (*p* ≤ 0.05).

Effects were found for treatment, week, and their interaction (*p* = 0.0005) for activity (m/s^2^). At Week 2, birds in the Green treatment were more active than all other treatments across both ages (261.17 ± 8.52 m/s^2^) (*p* < 0.001). Birds in the White treatment at Week 5 had the lowest activity (98.99 ± 8.52 m/s^2^) (*p* < 0.0001) compared to all other treatments across both ages (Figure 3).

### 3.6. Tibia Morphology

Tibia bone measures represent the average of the left and right tibia for each bird (Table 4). There was a trend found for the length of the tibia (*p* = 0.06), with birds in the Blue treatment (105.63 ± 1.58 mm) having longer tibias than birds in the White treatment (99.50 ± 1.58 mm). A tendency for the angle of the proximal head (*p* = 0.08) was found, with birds in the White treatment (39.49 ± 0.77°) having more obtuse proximal head angles than birds in the Green treatment (36.83 ± 0.77°).

### 3.7. Processing Performance

Body weights on D42 were not different across treatments, averaging 3292 g. There was no treatment effect on the occurrence of WB for scores 0 (68.25%), 1 (23.44%), or 2 (8.31%) (data not shown). Treatment differences were found with parts yields of fat pad (*p* = 0.02), breast (*p* = 0.01), tenders (*p* = 0.003), leg quarters (*p* = 0.01), rack and skin (*p* < 0.0001), white meat (breast and tenders) (*p* = 0.001), and carcass (*p* = 0.02) (Table 5). Birds in the Green treatment had lower fat pad yields (1.33 ± 0.02%) than birds in the Blue (1.43 ± 0.02%) (*p* = 0.01) and White treatments (1.42 ± 0.02%) (*p* = 0.02). Birds in the White treatment had greater breast yields (26.89 ± 0.13%) than birds in the Green (26.39 ± 0.13%) (*p* = 0.008) and birds in the Blue treatment (26.41 ± 0.13%) (*p* = 0.01). Birds in the White treatment had greater white meat yields (31.89 ± 0.14%) than birds in the Green (31.33 ± 0.14) (*p* = 0.005) and Blue treatments (31.21 ± 0.14%) (*p* = 0.0009). Birds in the Blue treatment had lower carcass yields (72.34 ± 0.17%) than birds in the Green (72.95 ± 0.17%) (*p* = 0.01) and White treatment (72.85 ± 0.17%) (*p* = 0.03). Birds in the Blue treatment had lower tender yields (4.87 ± 0.02%) than birds in the Green (4.94 ± 0.02%) (*p* = 0.04) and White treatments (5.00 ± 0.02%) (*p* = 0.0008). Birds in the Green treatment had greater leg quarters yield (30.90 ± 0.10%) than birds in the Blue treatment (30.48± 0.10%) (*p* = 0.004). Birds in the Blue treatment had greater rack and skin yield (27.02 ± 0.09%) than birds in the White (26.45 ± 0.09%) (*p* < 0.0001) and Green treatments (26.67 ± 0.09%) (*p* = 0.005).

## 4. Discussion

This study comprehensively evaluated the effects of white, blue, and green lighting on broiler chickens by measuring a wide range of behavioral, physiological, anatomical, and production outcomes. Broiler activity levels, fear and stress responses, tibia morphology, performance, blood cell profiles, and processing performance were measured. While previous research has examined the effects of colored lighting on some of these measures, few studies have integrated all these measures within the same study. This holistic approach provides a more complete understanding of how lighting environments affect broiler welfare and productivity.

Although activity and parts yields were affected by light color, body weight and feed efficiency (FCR) were not affected. These results highlight the importance of integrating both welfare and performance outcomes concurrently to ensure that improvements in welfare are not achieved at the expense of production efficiency. Similar results have been reported; for example, Prayitno et al. [11] found no significant effects of colored lighting (white, blue, green, and red) on body weight or FCR. Prayitno et al. [11] raised 40 male and 40 female broilers in light wavelength treatments to assess behavior, performance, and bone health. The authors suggested that their sample size may have been insufficient to detect effects of lighting on body weight; however, they observed greater skin and bone weights in green and blue light. These results agreed with the current study, where rack and skin yields were also greater in birds raised under blue light. These results suggest that light color may influence how body mass is distributed rather than total growth. It is possible that more pronounced effects could have been detected if body composition had been measured at earlier ages, allowing for a more detailed growth pattern over time.

Other studies have reported stage-specific effects of colored lighting on broilers. For example, Cao et al. [15] found that both green and blue light increased body weight from day 6 to day 18 and blue light increased body weight from day 38 to day 48. These growth patterns may be attributed to increased skeletal muscle cell proliferation during early development [32], in which short wavelengths may penetrate soft tissue and bone more effectively than mature, developed bone [33]. However, tibia morphology was largely unaffected by light wavelength in the current study, consistent with results reported by others [34,35].

Broiler processing yields can be impacted by multiple lighting factors, including photoperiod, intensity, and wavelength, but also the activity of the birds [36,37]. In the current study, green light increased bird activity, which also affected processing yields. Leg quarter yields were the greatest in green light, likely due to increased leg movement and muscle development associated with greater activity levels. Additionally, green light reduced abdominal fat pad yields, another outcome that may be attributed to increased activity. These results are consistent with Charles et al. [38], who reported that less active broilers had greater abdominal fat deposition. The relationship between broiler activity and parts yields has also been demonstrated in studies comparing slow-growing and fast-growing strains of broilers [36,39]. For example, Weimer et al. [39] and Fanatico et al. [34] found that broilers from slow-growing strains, which are typically more active, had greater leg quarters yields, while conventional fast-growing strains had greater breast yields. It was noted by Fanatico et al. [36] that this was due to increased foraging and movement. These patterns reinforce the link between activity and muscle development in specific body regions. In the present study, increased leg quarters yield may reflect the same relationship, suggesting that lighting can induce activity, which may shape some performance measures.

Performance measures relevant to industry are reflected in breast and white meat yields in the white light. Halevy et al. [32] subjected broilers to white, blue, green, and red light, and yields, weights, and satellite cell proliferation of the breast muscle were examined on day 35. The authors did not find differences in the breast yields, but found differences in the breast weights, with the green and blue treatments being the heaviest. The authors concluded that since satellite cells contribute to muscle growth, both green and blue light may stimulate early muscle development. Similarly, Cao et al. [15] found that broilers raised under blue light had the greatest breast muscle weight at day 49 compared to white, green, and red, which they linked to increased body weight. In contrast, the current study found that breast yield was the greatest in white light, which differs from both previous studies [15,32]. The differences may be due to the characteristics of the light used in the current study, which make direct comparisons difficult. Light intensity was measured at 0.1 W/m^2^ in Halevy et al. [32], but in the current study, light intensity was 0.03 to 0.04 W/m^2^. Additionally, Cao et al. [15] positioned lights 10 cm above the birds, whereas in the current study, lights were mounted at a height of 240 cm. Although light characteristics such as intensity, distance, and spectral composition can be controlled, subtle variations may contribute to inconsistent results in growth and processing performance.

Latency to right during the TI test was not affected by light color, which contrasts findings from previous studies [5,20]. In the current study, birds in blue light had numerically shorter latencies to right, although these differences were not statistically significant. Researchers hypothesized that blue light reduced activity and fear responses in broilers. Franco et al. [5] found that birds raised in blue light had the shortest latency to right during the TI test, indicating a reduction in fear. The authors attributed this response to the calming effects of blue light. In the current study, reduced activity was observed at two weeks of age in broilers under blue light, but this did not translate to any significant reductions in fear responses. While blue light may be effective at reducing fear responses in broilers in some studies, its effects on activity warrant further investigation.

Immune health in broilers is affected by lighting wavelengths [24]. In the current study, broilers under white light had greater circulating concentrations of lymphocytes, specifically T cells, compared to those under blue light, though not significantly different from green light. Xie et al. [24] found that white and green light increased T cell proliferation in vitro, suggesting improved cellular immune function. These results, along with findings from the current study, suggest that green and white light may enhance T cell-related immune responses compared to blue light, which may be indicative of improved adaptive immune function [15,24].

Infrared thermography was used as a noninvasive measure of stress in the current study, but no differences were observed. This may be due to the lack of environmental stressors applied to the birds, similar to why other physiological stress measures (e.g., CORT, HLR, and relative bursa weights) were not different. While Franco et al. [5] found reduced HLRs in birds under blue light at day 22 without a challenge or stressor applied, the current study did not replicate these results. This demonstrates the lack of correspondence in immune health (T cells and lymphocytes) to physiological measures, which may suggest there is not a strong relationship in broilers. These discrepancies may highlight the sensitivity of the stress response to experimental conditions and the need for standardized methodologies in lighting research.

Light wavelength has been shown to have an impact on behavioral responses, as evidenced through current and previous studies [11,40]. In the present study, birds under green light were more active than those under white or blue light. This contrasts findings of Prayitno et al. [11], who found broilers in white and red light were the most active, while those in green light walked the least. Behavioral differences with white light across studies may stem from the broad spectrum of white light, as it emits both long and short wavelengths [8]. Additionally, the methodology for measuring lighting or behavior across studies could generate inconsistent results. Similarly, Khosravinia [40] found that broilers raised under yellow, orange, red, or green light preferred green light as they walked and spent more time under it. Red light, known for its deeper tissue penetration and hypothalamic stimulation [41], was not included in the current study due to its potential to modulate endocrine responses. While green light may be preferable to broilers and increase activity, other studies have found white and red light increase activity, suggesting that the effects of light on broiler behavior are complex and not yet fully understood.

LEDs are commonly used in commercial poultry houses and offer advantages in energy efficiency and spectral control [15,42,43,44,45,46], but variability in light source type, flicker rate, and measurement methods can contribute to inconsistent results across studies [45]. For example, Prayitno et al. [11] used tungsten filament bulbs with colored filters, while the current study used light-emitting diodes. Additionally, the flicker rate of light, which poultry can perceive as low as 100 Hz, may influence activity levels [8,47]. While Prayitno et al. [11] used 5 min scan sampling to observe behavior, the current study measured activity through activity loggers. Both methods of behavioral observation provide valuable information about behavior, but direct comparisons between them may be limited in accuracy due to differences in methodology and data interpretation. Lighting treatment effects can also conflict if lighting specifications are not controlled, so direct comparisons across studies using different sampling techniques and lighting should be made with caution.

## 5. Conclusions

The objective of this study was to examine the effect of white, blue, and green light on broiler production performance, behavior, tibia morphology, blood cell profiles, and stress indicators. Broilers in white light were hypothesized to have increased activity, lower performance, and greater fear and stress responses than birds in blue and green light, but results did not support this hypothesis. Overall, light color had no significant effect on production, fear and stress levels, and tibia morphology. However, the color of light impacted activity, immune cells, and performance yields.

Green light had positive effects by promoting activity, particularly among younger broilers, which corresponded to increased leg quarters yield and fat pad yields. Broilers under white light were less active but had higher breast and white meat yields. These differences suggest that producers may prefer white light over green depending on the market demands and performance priorities. Blue light lowered adaptive immune cells, which may indicate compromised health for broilers under monochromatic blue lighting. These findings suggest that light color can be strategically used to target welfare or production goals.

## Figures and Tables

**Table 1:** Body weight (BW) and feed conversion ratio (FCR) of broilers raised under White, Blue, and Green light at bi-weekly diet phase changes (Starter, Grower, and Finisher).

Measures	White	Blue	Green	SEM	*p*-Value
**BW (kg) ^1^**					
Starter (D14)	0.49	0.47	0.48	0.01	0.12
Grower (D28)	1.90	1.86	1.88	0.01	0.06 *
Finisher (D41)	3.30	3.32	3.32	0.03	0.57
**FCR ^2^**					
Starter (D14)	1.20	1.23	1.22	0.01	0.32
Grower (D28)	1.41	1.43	1.44	0.02	0.54
Finisher (D41)	1.89	1.86	1.88	0.03	0.75
Cumulative	1.57	1.58	1.59	0.01	0.69

^1^ Body weights were taken at the pen level and calculated by dividing the total pen weight by the number of birds in the pen. ^2^ Feed conversion ratios were calculated by adjusting for mortalities that occurred within the diet phases. * Tendency for 0.05 < *p* ≤ 0.10.

**Table 2:** Concentrations (10^3^ cells/µL) of peripheral blood heterophils, lymphocytes, T cells, B cells, thrombocytes, WBC, and RBC, and HLR and TBR in 21- and 41-day-old broilers raised under White, Blue, and Green light.

	HLR ^1,2^	Heterophils (10^3^/uL)	Lymphocytes (10^3^/uL)	TBR ^3^	T Cells (10^3^/uL)	B Cells (10^3^/uL)	Thrombocytes (10^3^/uL)	WBC (10^3^/uL)	RBC (10^3^/uL)
**Treatment**									
White	0.43	4.28	10.28 ^a^	5.65	8.64 ^a^	1.64	39.83	19.39	2426.80
Blue	0.49	4.03	8.62 ^b^	5.48	7.16 ^b^	1.46	33.41	18.86	2410.63
Green	0.45	4.32	9.83 ^ab^	5.27	8.16 ^ab^	1.67	34.11	19.78	2390.36
SEM	0.03	0.26	0.40	0.30	0.33	0.10	2.31	0.70	37.43
**Age (Days)**									
21	0.46	3.24 ^b^	7.38 ^b^	5.83 ^a^	6.23 ^a^	1.15 ^a^	35.80	16.17 ^b^	2194.70 ^b^
41	0.45	5.17 ^a^	11.77 ^a^	5.09 ^b^	9.74 ^b^	2.02 ^b^	35.76	22.52 ^a^	2623.83 ^a^
SEM	0.02	0.21	0.33	0.25	0.27	0.08	1.92	0.57	30.50
**Treatment × Age**									
White × 21	0.41	3.10	7.75	5.95	6.61	1.15	40.49	16.26	2190.27
White × 41	0.46	5.45	12.	5.35	10.68	2.13	39.17	22.52	2663.34
Blue × 21	0.49	3.30	6.93	5.99	5.87	1.06	33.14	15.96	2222.91
Blue × 41	0.49	4.77	10.32	4.97	8.45	1.86	33.67	21.76	2598.36
Green × 21	0.46	3.35	7.48	5.56	6.22	1.25	33.78	16.28	2170.93
Green × 41	0.44	5.28	12.18	4.98	10.09	2.08	34.44	23.28	2609.80
SEM	0.04	0.37	0.57	0.43	0.47	0.14	3.22	0.99	52.88
***p*-values**									
Treatment	0.44	0.72	0.01	0.69	0.009	0.32	0.11	0.64	0.79
Age	0.73	0.0001	0.0001	0.04	0.0001	0.0001	0.98	0.0001	0.0001
Treatment × Age	0.71	0.54	0.32	0.85	0.25	0.81	0.94	0.83	0.65

^1^ Abbreviations: HLR, heterophil-to-lymphocyte ratio; TBR, T cell and B cell ratio; WBC, white blood cell; RBC, red blood cell. All cell concentrations were analyzed using flow cytometry. ^2^ The HLR was calculated by dividing the concentration of heterophils by the concentration of lymphocytes. ^3^ The TBR was calculated by dividing the concentration of T cells by the concentration of B cells. ^a,b^ Means not sharing the same letter within the same column for each effect indicates a significant difference (*p* ≤ 0.05).

**Table 3:** Thermal image surface temperatures of the right eye and beak (°C) in broilers raised under white, blue, and green light at D41.

Temperature (°C) ^1^	White	Blue	Green	SEM	*p*-Value
**Eye ^2^**					
Minimum	30.10	30.59	30.94	0.28	0.11
Average	30.88	31.30	31.76	0.27	0.09 *
Maximum	31.74	32.20	32.67	0.28	0.07 *
**Beak**					
Minimum	31.39	30.09	32.10	0.76	0.18
Average	33.05	31.50	33.49	0.78	0.18
Maximum	34.41	32.67	34.67	0.80	0.18

^1^ Thermal images were collected using a thermal camera that was 25.40 to 30.48 cm away from the bird. Images were later analyzed using SmartView software by selecting the same-sized area on the right eye and beak for each bird and using the minimum, average, and maximum temperature (°C). ^2^ Thermal images were always taken of the right eye and right side of the beak for each bird. * Tendency for 0.05 < *p* ≤ 0.10.

**Table 4:** Tibiotarsus (tibia) morphology of broilers raised under White, Blue, and Green light at D41. Data represent the average of the left and right tibias.

Measure ^1^	White	Blue	Green	SEM	*p*-Value
Length (mm)	99.5	105.63	101.75	1.58	0.06 *
PHW (mm)	28.26	28.38	28.62	0.48	0.87
Angle (°)	39.49	38.92	36.83	0.77	0.08 *
DHW (mm)	21.3	21.59	21.78	0.44	0.75
90% (mm)	25.14	25.33	25.57	0.72	0.92
75% (mm)	14.08	13.96	14.54	0.45	0.65
50% (mm)	10.21	9.77	10.70	0.35	0.23
25% (mm)	12.71	12.98	13.16	0.38	0.71
10% (mm)	18.89	18.84	19.12	0.56	0.93
MID (mm)	1.34	1.70	1.64	0.14	0.20
LID (mm)	1.52	1.74	1.75	0.15	0.49
DID (mm)	5.88	5.95	6.02	0.15	0.81
Ash (g)	3.62	3.68	3.85	0.22	0.75
Ash (%) ^2^	43.90	45.72	44.67	0.53	0.31

^1^ Abbreviations: DHW, distal head width; DID, distal intercondylar groove depth; LID, lateral intercondylar groove depth; MID, medial intercondylar groove depth; PHW, proximal head width. All measurements except ash (%) were adapted from procedures in Tijare et al. [31]. ^2^ Bone ash (%) was calculated by dividing the ash mass (g) by the bone mass (g) and multiplying it by 100. * Tendencies for 0.05 < *p* ≤ 0.10.

**Table 5:** Body weight (g) and processing yields (%) for 42-day-old broilers raised under White, Blue, and Green light. Part yields were calculated from the chilled carcass weights.

Yield (%) ^1^	White	Blue	Green	SEM	*p*-Value
Carcass	72.85 ^a^	72.34 ^b^	72.95 ^a^	0.17	0.02
Breast	26.89 ^a^	26.41 ^b^	26.39 ^b^	0.13	0.01
Tenders ^2^	5.00 ^a^	4.87 ^b^	4.94 ^a^	0.02	0.003
White Meat ^2^	31.89 ^a^	31.21 ^b^	31.33 ^b^	0.14	0.001
Wings	10.28	10.36	10.25	0.03	0.07 *
Leg Quarters	30.62 ^ab^	30.48 ^b^	30.90 ^a^	0.10	0.01
Rack + Skin	26.45 ^b^	27.02 ^a^	26.67 ^b^	0.09	<0.0001
Fat Pad	1.41 ^a^	1.43 ^a^	1.33 ^b^	0.02	0.02
Body Weight (g) ^3^	3287.2	3299.6	3288.3	27.81	0.94

^1^ All birds were processed after a 10 h feed withdrawal, and parts yields were calculated from chilled carcass weights. ^2^ White meat was the combination of breast and tenders. ^3^ Body weights were taken directly prior to the birds being hung on shackles. ^a,b^ Means not sharing the same letter across columns differ at *p* ≤ 0.05. * Tendencies for 0.05 < *p* ≤ 0.10.

## Data Availability

The authors will provide the data supporting the results upon request.
